# Transparency and completeness of reporting of depression screening tool accuracy studies: A meta‐research review of adherence to the Standards for Reporting of Diagnostic Accuracy Studies statement

**DOI:** 10.1002/mpr.1939

**Published:** 2022-09-01

**Authors:** Elsa‐Lynn Nassar, Brooke Levis, Marieke A. Neyer, Danielle B. Rice, Linda Booij, Andrea Benedetti, Brett D. Thombs

**Affiliations:** ^1^ Lady Davis Institute for Medical Research Jewish General Hospital Montreal Quebec Canada; ^2^ Department of Psychiatry McGill University Montreal Quebec Canada; ^3^ Centre for Prognosis Research School of Medicine Keele University Staffordshire UK; ^4^ Department of Psychology McGill University Montreal Quebec Canada; ^5^ Department of Psychology Concordia University Montreal Quebec Canada; ^6^ CHU Sainte‐Justine Hospital Research Centre Montreal Quebec Canada; ^7^ Department of Epidemiology, Biostatistics, and Occupational Health McGill University Montreal Quebec Canada; ^8^ Department of Medicine McGill University Montreal Quebec Canada; ^9^ Respiratory Epidemiology and Clinical Research Unit McGill University Health Centre Montreal Quebec Canada; ^10^ Department of Educational and Counselling Psychology McGill University Montreal Quebec Canada; ^11^ Biomedical Ethics Unit McGill University Montreal Quebec Canada

**Keywords:** depression, diagnostic test accuracy, reporting guidelines, screening, STARD

## Abstract

**Objectives:**

Accurate and complete study reporting allows evidence users to critically appraise studies, evaluate possible bias, and assess generalizability and applicability. We evaluated the extent to which recent studies on depression screening accuracy were reported consistent with Standards for Reporting of Diagnostic Accuracy Studies (STARD) statement requirements.

**Methods:**

MEDLINE was searched from January 1, 2018 through May 21, 2021 for depression screening accuracy studies.

**Results:**

106 studies were included. Of 34 STARD items or sub‐items, the number of adequately reported items per study ranged from 7 to 18 (mean = 11.5, standard deviation [SD] = 2.5; median = 11.5), and the number inadequately reported ranged from 3 to 17 (mean = 10.1, SD = 2.5; median = 10.0). There were eight items adequately reported, seven partially reported, 11 inadequately reported, and four not applicable in ≥50% of studies; the remaining four items had mixed reporting. Items inadequately reported in ≥70% of studies related to the rationale for index test cut‐offs examined, missing data management, analyses of variability in accuracy results, sample size determination, participant flow, study registration, and study protocol.

**Conclusion:**

Recently published depression screening accuracy studies are not optimally reported. Journals should endorse and implement STARD adherence.

## 1 INTRODUCTION

Major depression is a common and disabling disorder that accounts for more years of healthy life lost than any other medical condition (Lopez et al., 2006; Mathers et al., 2006; Moussavi et al., 2007; Whiteford et al., 2013). Depression screening has been proposed to identify individuals with unrecognized and untreated depression (Siu et al., 2016). Screening typically involves using depression symptom questionnaires to classify individuals as positive or negative screens based on scoring above or below a cut‐off. Those above the cut‐off can be interviewed to determine if they have major depression, whereas those below the cut‐off are not further assessed. Whether screening should be implemented, however, is controversial, and depression screening guidelines and policies vary substantially (Thombs et al., 2017, 2021). For example, the United States Preventive Services Task Force has recommended screening for depression in general adult and perinatal populations (Siu et al., 2016). In contrast, the United Kingdom National Screening Committee (UK National Screening Committee, n.d.) and the Canadian Task Force on Preventive Health Care (Joffres et al., 2013) have recommended against depression screening due to a lack of direct evidence from trials that screening improves health outcomes and due to concerns about resource consumption and possible harms.

For depression screening to improve upon routine care, screening tools must accurately identify individuals not currently in treatment or seeking treatment and whose depression would not otherwise be recognized by a health care provider (Joffres et al., 2013; Nassar et al., 2022b; Rice & Thombs, 2016; Thombs et al., 2011, 2012). Thus, well‐conducted and reported studies that evaluate the accuracy of depression screening tools are needed. Thorough and accurate reporting of design, methods, and results of studies allows users of evidence to critically appraise reports, evaluate the potential for bias (internal validity), and assess generalizability and applicability (external validity). Many study reports on test accuracy, however, fail to describe core elements transparently and completely, such as participants included, study design, and actual study results (Bastos et al., 2020; Carpenter et al., 2019; Korevaar et al., 2014).

Reporting guidelines have been developed to facilitate critical appraisal and interpretation of biomedical research by providing guidance to authors on a minimal set of items that should be reported (The EQUATOR Network, n.d.). Indeed, reporting guidelines have been shown to improve the completeness and transparency of reports of trials and diagnostic accuracy studies (Cobo et al., 2011; Korevaar et al., 2015; Smidt et al., 2006; Tunis et al., 2013; Turner et al., 2012). The Standards for Reporting of Diagnostic Accuracy Studies statement (STARD) statement consists of a 30‐item checklist that reflects a minimum set of information that should be included in reports of diagnostic accuracy (Bossuyt et al., 2003; Cohen et al., 2016). Items address reporting in the title and abstract, introduction, methods, results, discussion, and other information. No study has assessed the extent to which studies on the diagnostic accuracy of depression screening tools have reported results consistent with the STARD recommendations.

The objective of the present study was to review recently published studies of depression screening tool accuracy to assess the proportion of studies that adequately reported, partially reported, or inadequately reported each item of the STARD checklist (Cohen et al., 2016).

## 2 METHODS

This was part of a series of three meta‐research reviews that evaluated recently published studies of depression screening tool accuracy. The other two reviews examined the reporting of sample size calculations and precision of accuracy estimates (Nassar et al., 2022a) and the characteristics of participants included in studies (Nassar et al., 2022b). Prior to initiating the present study, a study protocol was posted on the Open Science Framework (https://osf.io/5tvf3/).

### 2.1 Eligibility

Primary studies in any language were eligible if they reported sensitivity and specificity estimates for one or more depression screening tools compared to depression classification based on a diagnostic interview. Primary studies were excluded if the reference standard was based on chart notes or a score above a threshold on another self‐report measure or rating scale. Primary studies that included only individuals seeking or receiving mental health services were also excluded since screening is conducted to identify individuals with unrecognized depression (Nassar et al., 2022b; Rice & Thombs, 2016; Thombs et al., 2011, 2012, 2021).

### 2.2 Identification of recently published primary studies

We searched MEDLINE (PubMed interface) on May 21, 2021 for primary studies published January 1, 2018, or later, using the search terms (depress*[Title/Abstract] AND sensitivity [Title/Abstract] AND specificity [Title/Abstract]) AND (“2018/01/01” [Date—Publication]: “3000” [Date—Publication]), restricted to title or abstract. We restricted the search to MEDLINE because a previous study found that 94% of a set of 234 studies included in 16 meta‐analyses of depression screening tools published between 2005 and 2014 were indexed in MEDLINE (Rice et al., 2016). Since the purpose of the present study was to evaluate a representative sample of studies, but not to necessarily uncover all published studies, and since searching additional databases requires additional resources, we did not include other databases. We included studies published in 2018 or later to examine recent studies reflecting relatively current practices. The PubMed search was conducted via DistillerSR (Evidence Partners), and citations were uploaded to the platform. Two investigators independently reviewed studies for eligibility. If either reviewer deemed a study potentially eligible based on title and abstract review, full‐text review was conducted, also independently by two reviewers. Any disagreements after full‐text review were resolved by consensus.

### 2.3 Data extraction

For all data extraction, one reviewer extracted the data from each included study, and a second reviewer verified the extracted data using the DistillerSR Quality Control function. Any discrepancies were resolved by consensus with a third reviewer consulted if necessary.

We extracted information using a standardized data extraction form via DistillerSR. For each primary study, we extracted (1) the first author's last name; (2) the publication year; (3) the journal and its most recent impact factor prior to or including the publication year; (4) the country; (5) the screening tool(s) evaluated and the reference standard; (6) the study population; (7) the number of participants; and (8) the number of depression cases.

### 2.4 Evaluation of completeness and transparency of reporting

We evaluated the completeness and transparency of reporting for all STARD items (Cohen et al., 2016). The STARD guidelines consist of 30 items. Four items have two parts, so there were 34 evaluations (Cohen et al., 2016). For each included study, reporting of each item was categorized as “adequately reported”, “partially reported”, “inadequately or not reported”, or “not applicable”. A coding manual was developed to ensure consistent and replicable assessment of reporting (see Supporting Information: Appendix 1) and was pilot tested in five diagnostic accuracy studies by four investigators to clarify wording and calibrate agreement. Assessment of completeness and transparency of reporting was then conducted by one reviewer and validated by a second reviewer. Any disagreements were resolved by discussion and consensus, with a third reviewer consulted as necessary.

We calculated the minimum, maximum, mean and standard deviation (SD), and median number of STARD items that were adequately reported across studies, as well as the numbers of items inadequately or not reported. Then, for each item, results were synthesized by totaling the number and percentage of studies adequately, partially, or inadequately reporting the item, among studies for which the item was applicable.

## 3 RESULTS

**FIGURE 1:**
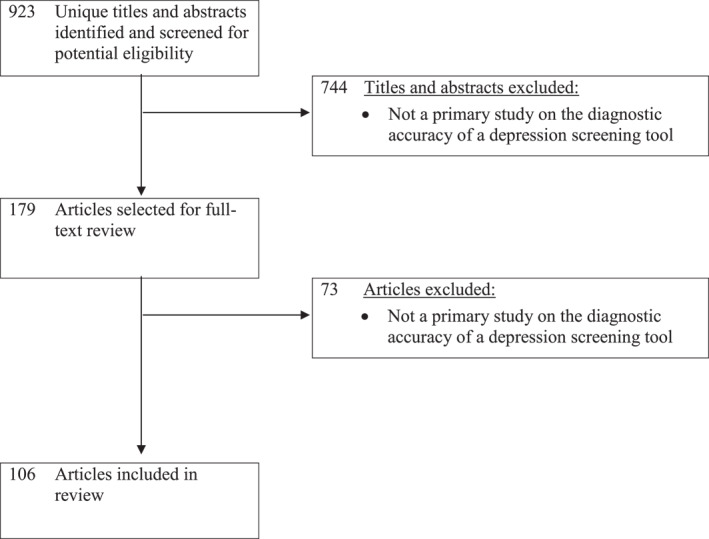
Flow diagram of selection of primary studies that evaluated the diagnostic accuracy of depression screening tools

The database search yielded 923 unique titles and abstracts. Of these, 744 were excluded after title and abstract review and 73 after full‐text review, leaving 106 eligible primary studies (Figure 1).

### 3.1 Characteristics of included screening accuracy studies

The 106 studies included sample sizes between 38 and 6700 (median = 243); number of depression cases ranged from 6 to 454 (median = 37). Most studies were from Asia (*n* = 32; 30%), Europe (*n* = 28; 26%), North America (*n* = 17; 16%), or Africa (*n* = 14; 13%). The most common depression screening tools were versions of the Patient Health Questionnaire (Kroenke et al., 2001; Spitzer et al., 1999) (35 studies), Geriatric Depression Scale (Yesavage et al., 1982) (9 studies), Edinburgh Postnatal Depression Scale (Cox et al., 1987) (8 studies), and Center for Epidemiologic Studies Depression Scale (Radloff, 1977) (7 studies). Seventy‐nine studies (75%) included samples of health care patients or users. There were 59 studies (56%) from journals with impact factor ≥3. See Table 1. Descriptions of included studies can be found in Supporting Information: Appendix 2.

**TABLE 1:** Included study characteristics

	Number (%) of primary studies (*N* = 106)
Publication year	
2018	33 (31%)
2019	35 (33%)
2020	25 (24%)
2021	13 (12%)
Journal impact factor	
0.6–1.9	11 (10%)
2.0–2.9	30 (28%)
3.0–4.9	51 (48%)
5.0–21.6	8 (8%)
Not available	6 (6%)
Continent	
Asia	32 (30%)
Europe	28 (26%)
North America	17 (16%)
Africa	14 (13%)
South America	9 (8%)
Australia/Oceania	6 (6%)
Depression screening tool	
Patient Health Questionnaire	35 (33%)
Geriatric Depression Scale	9 (8%)
Edinburgh Postnatal Depression Scale	8 (8%)
Center for Epidemiological Studies Depression	7 (7%)
Neurological Disorders Depression Inventory for Epilepsy	6 (6%)
Hospital Anxiety and Depression Scale	6 (6%)
Beck Depression Inventory	6 (6%)
Other^a^	29 (27%)
Study population	
Participants in health care settings	79 (75%)
General	15 (14%)
Neurologic disorders	13 (12%)
Pregnant/postpartum women	11 (10%)
Cancer	9 (8%)
Autoimmune diseases	6 (6%)
Cardiovascular diseases	6 (6%)
Human immunodeficiency virus	4 (4%)
Older adults	5 (5%)
Other^b^	10 (9%)
Participants in non‐health care settings^c^	27 (25%)

^a^ Patient Reported Outcomes Measurement Information System: *n* = 3; Hopkins Symptom Checklist: *n* = 2; Kessler Psychological Distress Scale: *n* = 2; Akena Visual Depression Inventory: *n* = 1; Anxiety, Depression And Mood Scale: *n* = 1; Brief Edinburgh Depression Scale: *n* = 1; Community Informant Detection Tool for Maternal Depression: *n* = 1; Connor‐Davidson Resilience Scale‐10: *n* = 1; Cornell Scale for Depression in Dementia: *n* = 1; Depression and Somatic Symptoms Scale—Depression Subscale: *n* = 1; Depression Inventory for Maintenance Hemodialysis Patients: *n* = 1; Depression Risk Questionnaire 7: *n* = 1; Distress Thermometer: *n* = 1; Emoticon scale: *n* = 1; Matthey Generic Mood Questionnaire: *n* = 1; Mood and Feeling Questionnaire: *n* = 1; No name: *n* = 1; Post‐stroke Depression Prediction Scale: *n* = 1; Postpartum Depression Predictors Inventory‐Revised: *n* = 1; Postpartum Depression Screening Scale: *n* = 1; Revised Children's Anxiety and Depression Scale: *n* = 1; Self‐Rating Depression Scale: *n* = 1; Whooley: *n* = 1; World Mental Health‐International College Student: *n* = 1; Zanmi Lasante Depression Symptom Inventory: *n* = 1.

^b^ Diabetes: *n* = 2; Hemodialysis: *n* = 2; Acne: *n* = 1; Bronchiectasis: *n* = 1; Chronic fatigue syndrome or myalgic encephalomyelitis: *n* = 1; Chronic low back pain: *n* = 1; Prenatal, postnatal, or general outpatients: *n* = 1; Traumatic brain injury: *n* = 1.

^c^Military Personnel: *n* = 2; University students: *n* = 2; Fathers of newborns: *n* = 1; Indigenous Australians: *n* = 1; Individuals in low literacy settings: *n* = 1; Individuals with intellectual disabilities: *n* = 1; Non‐professional caregivers: *n* = 1; Survivors of an airplane crash: *n* = 1.

### 3.2 Assessment of completeness and transparency of reporting

Of the 34 STARD items or sub‐items, the number of items adequately reported per study ranged from 7 to 18 (mean = 11.5, SD = 2.5; median 11.5), number inadequately reported from 3 to 17 (mean = 10.1, SD = 2.5; median = 10.0), and number partially reported from 4 to 14 (mean = 8.6, SD = 2.1; median = 8.0). See Supporting Information: Appendixes 3 and 4 for individual study results. As shown in Table 2, of the 34 STARD items, eight were adequately reported, seven were partially reported, and 11 were inadequately reported in ≥50% of studies; the remaining eight items were not applicable in most studies or had mixed reporting across studies.

**TABLE 2:** Results for completeness and transparency of reporting

			*N* = 106			
STARD 2015 checklist (section & topic)			Adequately reported *N* (%)	Partially reported *N* (%)	Inadequately or not reported *N* (%)	Not applicable *N* (%)
Title or abstract						
	1	Identification as a study of diagnostic accuracy using at least one measure of accuracy (such as sensitivity, specificity, predictive values, or AUC)	105 (99%)	0 (0%)	1 (1%)	0 (0%)
Abstract						
	2	Structured summary of study design, methods, results, and conclusions (for specific guidance, see STARD for abstracts)	16 (15%)	90 (85%)	0 (0%)	0 (0%)
Introduction						
	3	Scientific and clinical background, including the intended use and clinical role of the index test	93 (88%)	12 (11%)	1 (1%)	0 (0%)
	4	Study objectives and hypotheses	14 (13%)	86 (81%)	6 (6%)	0 (0%)
Methods						
Study design	5	Whether data collection was planned before the index test and reference standard were performed (prospective study) or after (retrospective study)	106 (100%)	‐‐‐‐‐‐‐‐‐‐‐‐^a^	0 (0%)	0 (0%)
Participants	6	Eligibility criteria	81 (76%)	11 (10%)	14 (13%)	0 (0%)
	7	On what basis potentially eligible participants were identified (such as symptoms, results from previous tests, inclusion in registry)	45 (42%)	12 (11%)	49 (46%)	0 (0%)
	8	Where and when potentially eligible participants were identified (setting, location and dates)	62 (58%)	41 (39%)	3 (3%)	0 (0%)
	9	Whether participants formed a consecutive, random or convenience series	50 (47%)	‐‐‐‐‐‐‐‐‐‐‐‐^a^	56 (53%)	0 (0%)
Test methods	10a	Index test, in sufficient detail to allow replication	27 (25%)	79 (75%)	0 (0%)	0 (0%)
	10b	Reference standard, in sufficient detail to allow replication	26 (25%)	78 (74%)	2 (2%)	0 (0%)
	11	Rationale for choosing the reference standard (if alternatives exist)	13 (12%)	35 (33%)	58 (55%)	0 (0%)
	12a	Definition of and rationale for test positivity cut‐offs or result categories of the index test, distinguishing pre‐specified from exploratory	26 (25%)	6 (6%)	74 (70%)	0 (0%)
	12b	Definition of and rationale for test positivity cut‐offs or result categories of the reference standard, distinguishing pre‐specified from exploratory	1 (1%)	0 (0%)	0 (0%)	105 (99%)
	13a	Whether clinical information and reference standard results were available to the performers/readers of the index test	2 (2%)	3 (3%)	8 (8%)	93 (88%)
	13b	Whether clinical information and index test results were available to the assessors of the reference standard	15 (14%)	42 (40%)	49 (46%)	0 (0%)
Analysis	14	Methods for estimating or comparing measures of diagnostic accuracy	103 (97%)	3 (3%)	0 (0%)	0 (0%)
	15	How indeterminate index test or reference standard results were handled	1 (1%)	‐‐‐‐‐‐‐‐‐‐‐‐^a^	2 (2%)	103 (97%)
	16	How missing data on the index test and reference standard were handled	14 (13%)	11 (10%)	81 (76%)	0 (0%)
	17	Any analyses of variability in diagnostic accuracy, distinguishing pre‐specified from exploratory	2 (2%)	14 (13%)	90 (85%)	0 (0%)
	18	Intended sample size and how it was determined	25 (24%)	5 (5%)	76 (72%)	0 (0%)
Results						
Participants	19	Flow of participants, using a diagram	5 (5%)	10 (9%)	91 (86%)	0 (0%)
	20	Baseline demographic and clinical characteristics of participants	86 (81%)	19 (18%)	1 (1%)	0 (0%)
	21a	Distribution of severity of disease in those with the target condition	30 (28%)	41 (39%)	35 (33%)	0 (0%)
	21b	Distribution of alternative diagnoses in those without the target condition	27 (25%)	42 (40%)	37 (35%)	0 (0%)
	22	Time interval and any clinical interventions between index test and reference standard	51 (48%)	‐‐‐‐‐‐‐‐‐‐‐‐^a^	55 (52%)	0 (0%)
Test results	23	Cross tabulation of the index test results (or their distribution) by the results of the reference standard	2 (2%)	103 (97%)	1 (1%)	0 (0%)
	24	Estimates of diagnostic accuracy and their precision (such as 95% confidence intervals)	7 (7%)	44 (42%)	55 (52%)	0 (0%)
	25	Any adverse events from performing the index test or the reference standard	0 (0%)	‐‐‐‐‐‐‐‐‐‐‐‐^a^	0 (0%)	106 (100%)
Discussion						
	26	Study limitations, including sources of potential bias, statistical uncertainty, and generalizability	29 (27%)	71 (67%)	6 (6%)	0 (0%)
	27	Implications for practice, including the intended use and clinical role of the index test	105 (99%)	‐‐‐‐‐‐‐‐‐‐‐‐^a^	1 (1%)	0 (0%)
Other information						
	28	Registration number and name of registry	3 (3%)	‐‐‐‐‐‐‐‐‐‐‐‐^a^	103 (97%)	0 (0%)
	29	Where the full study protocol can be accessed	0 (0%)	‐‐‐‐‐‐‐‐‐‐‐‐^a^	106 (100%)	0 (0%)
	30	Sources of funding and other support; role of funders	42 (40%)	53 (50%)	11 (10%)	0 (0%)

Abbreviations: AUC, area under the curve; STARD, standards for reporting of diagnostic accuracy studies statement.

^a^ There were no coding options for these fields.

Of the four items in the Title, Abstract, and Introduction sections, two items were adequately reported in most studies (identifying the study as a diagnostic accuracy study, 99%; providing a scientific and clinical background, 88%), while two items, which related to the abstract and study objectives, were partially reported in most studies (85% and 81%, respectively).

Of 17 items or sub‐items in the Methods section, one item related to aspects of study design, four to participants, seven to test methods, and five to analysis. Among the five study design and participant items, three items were adequately reported in most studies (planning of data collection, 100%; eligibility, 76%; sites and dates of recruitment, 58%), while one item, which related to sampling, was inadequately reported in 53% of studies. Among the seven test methods items, two items were partially reported in most studies (description of index test, 75%; description of reference standard, 74%), and two items were inadequately reported in most studies (rationale for choosing the reference standard, 55%; rationale for index test cut‐offs examined, 70%). Among the five analysis items, one item was adequately reported in most studies (diagnostic accuracy estimation methods, 97%), and three items were inadequately reported in most studies (handling of missing data, 76%; description of analyses of variability, 85%; sample size determination, 72%).

Of the eight items in the Results section, five related to aspects of participants and three to aspects of test results. Among the five participant items, one item was adequately reported in most studies (baseline demographic and clinical characteristics, 81%), while two items were inadequately reported in most studies (diagram to describe the flow of participants through studies, 86%; time interval between index test and reference standard administration, 52%). Among the three items related to test results, one was partially reported in most studies (cross‐tabulation of index test by reference standard results, 97%), and one was inadequately reported in most studies (estimates of diagnostic accuracy with their precision, 52%).

Of the two items in the Discussion section, one was adequately reported in most studies (implications for practice, 99%), and one was partially reported in most studies (study limitations, 67%).

Of the three items in the Other Information section, two items were inadequately reported in most studies (study registration, 97%; study protocol, 100%), while one item was partially reported in 50% of studies (sources of funding and role of funders).

## 4 DISCUSSION

We evaluated the degree to which 106 recently published primary studies that assessed the accuracy of depression screening tools reported results consistent with the set of minimal reporting requirements described in the STARD reporting guideline (Cohen et al., 2016). Overall, we found that recently published diagnostic accuracy studies on depression screening tools are not optimally reported, with individual studies adequately reporting an average of approximately a third of the items in the STARD checklist. Of the 34 STARD items and sub‐items, eight were adequately reported, seven were partially reported, 11 were inadequately reported, and four were not applicable in at least half of included studies; the remaining four items had mixed reporting across studies. Overall, most studies adhered to the STARD reporting guideline for the title, abstract, introduction, and discussion, and most items in these sections were reported fully or partially adequately by the majority of studies. However, included studies did not optimally report elements related to methods, results, and other information sections. For the Methods section, most studies did not optimally report items related to the sampling method (53% inadequate), the rationale for choosing the reference standard (55% inadequate), and the rationale for choosing the selected cut‐offs (70% inadequate). For the analysis subsection of the Methods section, of the four items applicable in most studies, only one item was adequately reported in most studies (methods for estimating diagnostic accuracy; 97% adequate), whereas three items, which related to the management of missing data (76% inadequate), description of any analysis of variability in diagnostic accuracy (85% inadequate), and targeted sample size (72% inadequate), were inadequately reported. For the Results section, most studies did not report the flow of participants using a diagram (86% inadequate), the time interval between the administration of the index test and the reference standard (52% inadequate), and the estimates of diagnostic accuracy with their precision (52% inadequate). Authors did not adequately report elements related to the Other Information section, with most studies inadequately reporting the study registration (97% inadequate) and study protocol (100% inadequate).

There are several notable reporting gaps. First, most studies did not provide a rationale for index test positivity cut‐offs that were reported or report estimates of accuracy for all assessed cut‐offs. Selectively reporting results for only some cut‐offs that were assessed, usually the most accurate cut‐offs, is a common practice in depression screening studies that has been previously described and shown to result in overly optimistic conclusions about the accuracy of depression screening tests (Levis et al., 2017). Second, screening accuracy studies should be conducted with sample sizes with adequate numbers of participants with and without depression to generate reasonably precise estimates of sensitivity (the proportion of participants with depression correctly identified by the screening tool) and specificity (the proportion of those without depression correctly ruled out by the screening tool), as well as other outcomes. Specifically, to ensure that studies generate reasonably precise estimates of sensitivity and specificity, investigators should consider the precision that is needed for use in clinical practice and should calculate the sample size required to achieve that level of precision. However, we found that 72% of included studies did not specify the targeted sample size including how it was calculated. Similar trends were observed in reviews of depression screening tool accuracy studies published between (1) 2013 and 2015, where only three of 89 (3%) studies described a viable sample size calculation and 30 studies (34%) provided reasonably accurate confidence intervals around accuracy estimates (Thombs & Rice, 2016) and (2) 2018 and 2021, where only 12 of 106 (11%) studies described a viable sample size calculation and 36 (34%) provided reasonably accurate confidence intervals (Nassar et al., 2022a). Importantly, accuracy studies with small samples sizes often fail to identify the most accurate cut‐off and overstate accuracy estimates for the cut‐offs they report (Bhandari et al., 2021). A simulation study based on real participant depression screening data from the Edinburgh Postnatal Depression Scale with over 13,000 total participants found that with simulated samples of 100 participants, study‐specific optimal cut‐offs that maximized combined sensitivity and specificity ranged from ≥5 to ≥17 compared to the true population optimal cut‐off of ≥11 (Bhandari et al., 2021). On average, simulated individual studies overestimated sensitivity by 6.5 and underestimated specificity by 1.3% points. In contrast, with samples of 1000 participants, study‐specific optimal cut‐offs ranged from ≥8 to ≥13; on average, sensitivity and specificity were overestimated and underestimated by 1.4% and 1.0% points, respectively (Bhandari et al., 2021). Third, reporting of missing data management and any performed analyses of variability in diagnostic accuracy, which is important in assessing possible bias and may have implications for study generalizability, was inadequately reported in most studies.

There were several important strengths to our study. First, this is the first study assessing the extent to which recently published studies on the accuracy of depression screening tools have adhered to the minimal reporting requirements described in the STARD reporting guideline. To our knowledge, no other study has assessed the extent to which any mental health screening accuracy studies have adhered to the STARD reporting guideline. Second, in developing a STARD coding manual prior to initiating this study, we ensured consistent and replicable assessment of reporting across coders and studies (see Supporting Information: Appendix 1). Third, we were able to include and code for all studies published since 2018 that were indexed in Medline, reflecting relatively current practices in depression screening accuracy studies.

The results of the present review should also be interpreted with respect to some limitations. First, we only searched the MEDLINE database for eligible studies, which could have led us to miss some eligible studies. However, searching only MEDLINE for studies of diagnostic test accuracy, generally, has been shown to not influence summary estimates in meta‐analyses (van Enst et al., 2014). In meta‐analyses on the accuracy of depression screening tools, specifically, restricting searches to MEDLINE has been shown to capture almost all (approximately 95%) the eligible studies (Rice et al., 2016). Thus, it is unlikely that our main findings would have changed substantively if other databases had been searched. Second, we did not extend our assessment to search for study protocols that were not cited or linked to included studies. Some authors may have included additional study details within the protocol. However, all included studies did not specify where the full study protocol can be accessed (100% inadequate), and unless protocols are linked to studies, readers would not have access to them. In addition, the STARD checklist is a minimum set of standards that should be adequately reported in studies of screening accuracy, irrespective of having been previously published in a protocol.

In summary, this study was the first to assess the completeness and transparency of reporting of diagnostic accuracy studies of depression screening tools. Recently published depression screening tool accuracy studies are not optimally reported. There is a need for attention to more fulsome reporting of methodological conduct of these studies, mostly related to test methods, test results, and analysis aspects. In order to improve the quality of reporting in depression screening accuracy studies, the research community, journal editors, reviewers, and funders should endorse and implement adherence to STARD.

## AUTHOR CONTRIBUTIONS

Elsa‐Lynn Nassar, Brooke Levis, Danielle B. Rice, Linda Booij, Andrea Benedetti, and Brett D. Thombs contributed to the conception and design of the study. Elsa‐Lynn Nassar, Brooke Levis, Marieke A. Neyer, and Brett D. Thombs contributed to data extraction, coding, and evaluation of included studies. Elsa‐Lynn Nassar, Brooke Levis, Andrea Benedetti, and Brett D. Thombs contributed to the data analysis plan, and Elsa‐Lynn Nassar conducted the analyses. Elsa‐Lynn Nassar drafted the manuscript, and Brooke Levis, Marieke A. Neyer, Danielle B. Rice, Linda Booij, Andrea Benedetti, and Brett D. Thombs provided critical reviews and approved submission of the final manuscript.

## CONFLICT OF INTEREST

All authors completed the ICJME uniform disclosure form and declared no support from any organization for the submitted work; no financial relationships with any organizations that might have an interest in the submitted work in the previous 3 years. All authors declare no other relationships or activities that could appear to have influenced the submitted work.

## Supporting information

Supporting Information 1

## Data Availability

The data that supports the findings of this study are available in the supplementary material of this article.
