# Supplementary material for: Transparency and completeness of reporting of depression screening tool accuracy studies: A meta‐research review of adherence to the Standards for Reporting of Diagnostic Accuracy Studies statement
Source: Int J Methods Psychiatr Res. 2022 Sep 1;32(1):e1939. doi: 10.1002/mpr.1939 (PMC9976600; doi:10.1002/mpr.1939)
Supplement: Supplementary file 1 — Supporting Information 1 [file MPR-32-e1939-s001.docx]

**Appendix 1. Coding Manual for Completeness and Transparency of Reporting**

| **STARD 2015 Checklist (section & topic)** | | | | **Adequately reported** | **Partially reported** | **Inadequately or not reported** | **Not applicable** |
| --- | --- | --- | --- | --- | --- | --- | --- |
| ***Title or abstract*** | | | | | | | |
|  | 1 | Identification as a study of diagnostic accuracy using at least one measure of accuracy  (such as sensitivity, specificity, predictive values, or AUC) | | Authors identified the study as a diagnostic accuracy study by describing it as a diagnostic (or screening) accuracy study or using at least one measure of accuracy | ------------ | Authors did not report either element |  |
| ***Abstract*** | | | | | | | |
|  | 2 | Structured summary of study design, methods, results, and conclusions  (for specific guidance, see STARD for Abstracts) | | Authors (1) provided a structured summary with objectives, methods, results, and conclusions, (2) reported number of participants with and without the target condition, and (3) an estimate of accuracy with precision (e.g., 95% confidence interval) | Authors only reported one element but not both | Authors did not report either element |  |
| ***Introduction*** | | | | | | | |
|  | 3 | Scientific and clinical background, including the intended use and clinical role of the index test | | Authors provided a scientific and clinical background, including (1) previous evidence on the topic or knowledge gaps including evidence on accuracy of test and (2) intended use or clinical role of the index test (e.g., screening, monitoring for relapse). Item must present (1) evidence related to test accuracy to situate study and (2) specifically mention role of test clinically | Authors only reported one element but not both | Authors did not report either element |  |
|  | 4 | Study objectives and hypotheses | | Authors described the study objective(s) or hypothesis(es), including the (1) index test, (2) reference standard including method (e.g., interview) and diagnostic target (e.g., major depression), and (3) at least one outcome (measure of accuracy) | Authors only reported one or two element(s) but not all three | Authors did not report any element |  |
| ***Methods*** | | | | | | | |
| Study design | 5 | Whether data collection was planned before the index test and reference standard were performed (prospective study) or after (retrospective study) | | It is clear whether data collection was planned before the index test and reference standard were performed (prospective study) or after (retrospective study) | ------------ | It is not clear whether data collection was planned before the index test and reference standard were performed (prospective study) or after (retrospective study) |  |
| Participants | 6 | Eligibility criteria | | Authors clearly described eligibility criteria for participation. If all patients in a care setting were eligible, this should be stated | Authors described some eligibility criteria or reasons some participants were excluded but not complete inclusion and exclusion criteria (e.g., not enough to replicate) or a statement that all patients were eligible | Authors did not describe any eligibility criteria for participation |  |
|  | 7 | On what basis potentially eligible participants were identified  (such as symptoms, results from previous tests, inclusion in registry)  *NOTE: Only consider items that would potentially need assessment/documentation about where the information was obtained (e.g., mental health diagnosis vs. age)* | | Authors clearly described on what basis potentially eligible participants were identified (such as symptoms, results from previous tests, inclusion in registry) | Authors provided some information but not a complete explanation (e.g., not enough to replicate) on what basis potentially eligible participants were identified | Authors did not describe on what basis potentially eligible participants were identified |  |
|  | 8 | Where and when potentially eligible participants were identified (setting, location and dates) | | Authors clearly described where and when potentially eligible participants were identified, including (1) setting (e.g., type of health care institution), (2) location (name of centre, city, or country), and (3) dates | Authors only reported one or two element(s) but not all three | Authors did not report any element |  |
|  | 9 | Whether participants formed a consecutive, random or convenience series | | Authors clearly described whether participants formed a consecutive, random, or convenience series | ------------ | Authors did not describe whether participants formed a consecutive, random, or convenience series |  |
| Test methods | 10a | Index test, in sufficient detail to allow replication | | Authors described the index test in sufficient detail to allow replication, including (1) version of test (if there are multiple versions), (2) language of test (if potentially different language from measure primary development language) and information on translation (if applicable) or citation to language validation source, and (3) administration of test (e.g., in paper, tablet, interviewer by phone or video) | Authors only reported one or two element(s) but not all three | Authors did not report any element |  |
|  | 10b | Reference standard, in sufficient detail to allow replication | | Authors describe the reference standard in sufficient detail to allow replication, including (1) diagnosis (e.g., major depression, any depressive disorder) and system (e.g., DSM, ICD), (2) interview methods used (e.g., SCID, CIDI, unstructured clinician interview) and interview version (if applicable), (3) language (if potentially not used in development language) and information on translation (if applicable), (4) qualifications or training of interviewers, and (5) administration format (e.g., in person, phone, video) | Authors only reported one to four element(s) but not all five | Authors did not report any element |  |
|  | 11 | Rationale for choosing the reference standard (if alternatives exist) | | Authors described the rationale (e.g., reliability, efficiency, accuracy when compared with alternative reference standards) for choosing the reference standard (if alternatives exist) | Authors described the rationale for choosing the reference standard (but not when compared with alternatives) | Authors did not describe the rationale for choosing the reference standard |  |
|  | 12a | Definition of and rationale for test positivity cut-offs or result categories of the index test, distinguishing pre-specified from exploratory  *NOTE: This item should be reported in the method section (not the results section)* | | Authors clearly described (1) which cut-off(s) were assessed and (2) the rationale for investigating this (these) cut-offs, distinguishing pre-specified from exploratory. If all possible cut-offs were assessed, a rationale is not necessary. | Authors only reported one element but not both | Authors did not report either element AND did not assess all possible cut-offs |  |
|  | 12b | Definition of and rationale for test positivity cut-offs or result categories of the reference standard, distinguishing pre-specified from exploratory | | Authors clearly described (1) the definition of and (2) rationale for test positivity cut-offs or results categories of the reference standard distinguishing pre-specified from exploratory | Authors only reported one or two element(s) of this item and not all three | Authors did not report any elements | This will only be applicable if a scoring system is used for determining classification (e.g., PSE interview) |
|  | 13a | Whether clinical information and reference standard results were available to the performers/readers of the index test  *NOTE: If the study population is not a clinical population, authors only need to report whether reference standard results were available to the performers/readers of the index test* | | Authors clearly identified whether (1) clinical information (e.g., previous test results, symptom reports) and (2) reference standard results were available to the performers/readers of the index test | Authors only reported one element but not both | Authors did not identify whether clinical information and reference standard results were available to the performers/readers of the index test | This will be not applicable for all self-report depression screening tools |
|  | 13b | Whether clinical information and index test results were available  to the assessors of the reference standard  *NOTE: If the study population is not a clinical population, authors only need to report whether index test results were available to the assessors of the reference standard* | | Authors clearly identified whether (1) clinical information (e.g., previous test results, symptom reports) and (2) index test results were available to the assessors of the reference standard | Authors only reported one element but not both | Authors did not report either element |  |
| Analysis | 14 | Methods for estimating or comparing measures of diagnostic accuracy | | Authors clearly reported the methods used for estimating diagnostic accuracy, including the measures used (e.g., sensitivity, specificity); if tests are compared, the authors should describe which statistical test(s) was/were performed and decision criteria (e.g., 95% confidence interval) | Authors reported some but not all applicable elements | Authors did not report any element |  |
|  | 15 | How indeterminate index test or reference standard results were handled | | Authors clearly described how indeterminate reference standard results were handled | ------------ | Authors did not describe how indeterminate index test or reference standard results were handled | This will not be applicable for most studies that use diagnostic interviews |
|  | 16 | How missing data on the index test and reference standard were handled | | Authors clearly reported how missing data on the (1) index test and (2) reference standard were handled | Authors only reported one element but not both | Authors did not report either element |  |
|  | 17 | Any analyses of variability in diagnostic accuracy, distinguishing pre-specified from exploratory | | Authors clearly (1) reported any analyses of variability in diagnostic accuracy, (2) distinguishing pre-specified from exploratory OR reported that no analyses of variability were conducted | Authors reported any analyses of variability in diagnostic accuracy, but did not distinguish pre-specified from exploratory OR did not report that no analyses of variability were conducted | Authors did not report any analyses of variability in diagnostic accuracy and did not indicate that they did not conduct any such analyses |  |
|  | 18 | Intended sample size and how it was determined | | Authors clearly identified (1) the intended sample size, (2) how it was determined, and (3) provided sufficient information to replicate. Authors must describe a viable sample size calculation (e.g., not justify the sample size based on past studies without a calculation) | Authors only reported one element but not both | Authors did not report either element |  |
| ***Results*** | | | | | | | |
| Participants | 19 | Flow of participants, using a diagram | | Authors clearly reported the flow of participants, using a diagram, including the numbers of participants who were (1) assessed for eligibility, (2) eligible, (3) received one or more index tests, and (4) received the reference standard with reasons provided if not. This information must be provided in the form of a diagram | Authors included a flow diagram, but it did not include all relevant items | Authors did not describe the flow of participants using a diagram |  |
|  | 20 | Baseline demographic and clinical characteristics of participants | | Authors clearly reported (1) baseline demographic and (2) clinical characteristics of participants. If the study population is not a clinical population, reporting of clinical characteristics is not required | Authors only reported one element but not both | Authors did not report either element |  |
|  | 21a | Distribution of severity of disease in those with the target condition | Authors clearly reported the distribution of severity of disease or the distribution of index test scores (e.g., mean, SD) in those with the target condition | | Authors reported the distribution of index test scores for the total screened sample, but not by cases and non-cases based on target condition separately | Authors did not report the distribution of severity of disease, or the distribution of index test scores (e.g., mean, SD) in those with the target condition |  |
|  | 21b | Distribution of alternative diagnoses in those without the target condition | Authors clearly reported the distribution of index test scores (e.g., mean, SD) in those without the target condition | | Authors reported the distribution of index test scores for the total screened sample, but not by cases and non-cases based on target condition separately | Authors did not report the distribution of index test scores (e.g., mean, SD) in those without the target condition |  |
|  | 22 | Time interval and any clinical interventions between index test and reference standard  *NOTE: If the time interval between the index test and reference standard was the same for all participants and reported in the methods (e.g., same day), then this item is coded as “adequately”* | Authors clearly indicated the time interval and described any clinical interventions between index test and reference standard (if not mentioned, assume there were none) | | ------------ | Authors did not indicate the time interval or describe any clinical interventions between index test and reference standard |  |
| Test results | 23 | Cross tabulation of the index test results (or their distribution)  by the results of the reference standard | Authors clearly reported the cross tabulation of the index test results (or their distribution) by the results of the reference standard for all cut-offs examined (e.g., 2x2 table) | | Authors reported the cross tabulation for some cut-offs examined or did not report cross tabulation but reported information to easily calculate | Authors did not report the cross tabulation of the index test results (or their distribution) by the results of the reference standard for any cut-offs examined (e.g., 2x2 table) and did not report information to easily calculate |  |
|  | 24 | Estimates of diagnostic accuracy and their precision (such as 95% confidence intervals) | Authors clearly (1) reported the estimates of diagnostic accuracy and (2) their precision (such as 95% confidence intervals) for all cut-offs examined. Authors must report estimates of diagnostic accuracy for all cut-offs examined. It must be clear which cut-offs were examined | | Authors reported the estimates of diagnostic accuracy for all cut-offs examined, but without their precision or did not report estimates for all cut-offs examined | Authors did not report the estimates of diagnostic accuracy for all cut-offs examined and did not report precision |  |
|  | 25 | Any adverse events from performing the index test or the reference standard | Authors clearly indicated any adverse events from performing the index test or the reference standard | | ------------ | Authors did not indicate any adverse events from performing the index test or the reference standard | This will likely not be applicable for most or all studies |
| ***Discussion*** | | | | | | | |
|  | 26 | Study limitations, including sources of potential bias, statistical uncertainty, and generalisability | Authors clearly identified study limitations, including (1) sources of potential bias, (2) statistical uncertainty (e.g., confidence intervals), and (3) generalisability | | Authors only reported one or two element(s) but not all three | Authors did not report any elements |  |
|  | 27 | Implications for practice, including the intended use and clinical role of the index test | Authors clearly identified the implications for practice, including the intended use and clinical role of the index test | | ------------ | Authors did not report how the text would be used in practice (e.g., screening) |  |
| ***Other Information*** | | | | | | | |
|  | 28 | Registration number and name of registry | Authors clearly reported the registration number and name of registry | | ------------ | Authors did not report the registration number and name of registry |  |
|  | 29 | Where the full study protocol can be accessed | Authors reported where the full study protocol can be accessed (e.g., citation or link) | | ------------ | Authors did not report where the full study protocol can be accessed with a citation or link |  |
|  | 30 | Sources of funding and other support; role of funders | Authors clearly reported (1) sources of funding and other support and (2) role of funders | | Authors only reported one element but not both | Authors did not report either element |  |

**Appendix 2. Primary Studies of the Diagnostic Accuracy of Depression Screening Tools**

| **First Author Last Name** | **Year** | **Journal** | **2020 Impact Factor** | **Country(ies)** | **Population** | ***N* Screened and Interviewed** | ***N* Depression Cases** | **Diagnostic  Criterion** | **Screening  Tool(s)** |
| --- | --- | --- | --- | --- | --- | --- | --- | --- | --- |
| Akena | 2018 | Br J Psychiatry | 9.3 | Uganda;  South Africa | Patients in low literacy settings | 343 | 78 | MINI | AViDI-18 |
| Alves | 2019 | Midwifery | 2.4 | Portugal | Perinatal women | 140 | 6 | SCID-IV | PDPI-R; EPDS |
| Arturo Cassiani-Miranda | 2021 | Gen Hosp Psychiatry | 3.2 | Colombia | Adult primary care patients | 243 | 51 | MINI | HADS-D |
| Aslan | 2020 | Front Psychiatry | 4.2 | Chile | Self-dependent primary care  outpatients aged 65 to 80 | 577 | 21 | CIDI | PHQ-9 |
| Baldellou Lopez | 2021 | J Psychosom Res | 3 | United Kingdom | Adults with dissociative seizures  in the previous 8 weeks | 368 | 114 | MINI | PHQ-9 |
| Ballester | 2019 | PloS One | 3.2 | Spain | First year university students  aged 18 to 24 | 287 | 26 | MINI | WMH-ICS online screening scale |
| Baumgartner | 2019 | Psychiatr Prax | 1.5 | Austria | Individuals aged 18 to 65 from the general population | 504 | 39 | SCAN | GDS-15 |
| Bautovich | 2018 | Australas Psychiatry | 1.4 | Australia | Patients aged over 18 years with  chronic haemodialysis | 45 | 6 | DSM-IV diagnosis by clinicians | BDI; CDI |
| Bernstein | 2018 | Inflamm Bowel Dis | 5.3 | United States | Individuals with inflammatory  bowel disease aged 18 years or older | 242 | 21 | SCID-IV | PHQ-2 ; PHQ-9; HADS-D ; PROMIS Depression |
| Bhana | 2019 | S Afr Med J | 1.6 | South Africa | Outpatients aged 18 years or older  attending primary healthcare clinics | 1206 | 57 | APC assessment by the primary healthcare nurse | PHQ-2 |
| Binagwaho | 2021 | BMC Pediatr | 2.1 | Rwanda | Children with HIV aged 7 to 14 | 296 | 42 | SCID-IV | CDST |
| Blanco | 2019 | Aging Ment Health | 3.7 | Spain | Non-professional caregivers | 294 | 30 | SCID-5 | CD-RISC 10 |
| Borghero | 2018 | Rev Med Chil | 0.6 | Chile | Adolescents aged 15 to 19 years | 245 | 210 | K-SADS-PL | PHQ-9 |
| Butnoriene | 2018 | BMC Psychiatry | 3.6 | Lithuania | Individuals aged 45 years or older with and without metabolic syndrome | 1115 | 201 | MINI | HADS-D |
| Cassiani-Miranda | 2021 | Rev Colomb Psiquiatr | NA | Colombia | Primary care patients aged 18 to 65 years | 243 | 53 | MINI | PHQ-9 |
| Chenneville | 2019 | J Affect Disord | 4.8 | United States | People with HIV aged 12 to 25 years receiving care in an integrated care setting | 121 | 37 | Mental health diagnostic interview | PHQ-9; CES-D |
| Clover | 2018 | Qual Life Res | 4.1 | Australia | Outpatients with cancer | 132 | 18 | SCID-IV | PROMIS-D-CAT; PROMIS-D-SF |
| Colomo | 2021 | Arch Bronconeumol | NA | Spain | Adult patients with bronchiectasis | 52 | 18 | MINI | HADS-D; BDI |
| Cruzado | 2018 | Support Care Cancer | 3.6 | Spain | Adult cancer patients | 130 | 6 | MINI (DSM-IV and the ICD-10) | HADS-D; DT; DEPQ |
| Cumbe | 2020 | BMC Psychiatry | 3.6 | Mozambique | Prenatal, postnatal, and general outpatients | 502 | 43 | MINI | PHQ-9 |
| Dajpratham | 2020 | BMC Psychiatry | 3.6 | Thailand | First-time stroke patients aged 45 and older | 115 | 23 | DSM-5 diagnosis by psychiatrist | PHQ-9 |
| Degefa | 2020 | BMC Psychiatry | 3.6 | Ethiopia | Adult outpatients with cancer | 163 | 25 | MINI | PHQ-9 |
| Durmaz | 2018 | North Clin Istanb | NA | Turkey | Older adults  (aged 65 or over) outpatients visiting a geriatric clinic | 329 | 81 | DSM-5 diagnosis | GDS-15; GDS-30 |
| Eriksen | 2019 | J Affect Disord | 4.8 | Norway | Home-dwelling adults aged 60 years or older | 194 | 56 | ICD-10 diagnosis | GDS-5; HADS-D |
| Figueiredo-Duarte | 2019 | Aging Ment Health | 3.7 | Portugal | Institutionalized adults aged 60 or older | 186 | 20 | MINI | GDS |
| Fuseekul | 2021 | Child Adolesc Psychiatry Ment Health | 3 | Thailand | Secondary school students aged 12-18 | 103 | 31 | K-SADS-PL (DSM-5) | MFQ |
| Gallis | 2018 | PeerJ | 3 | Pakistan | Community-based pregnant women | 1731 | 454 | SCID | PHQ-9 |
| Getting it Right Collaborative Group | 2019 | Med J Aust | 7.7 | Australia | Adults (18 years or older) identifying as Indigenous Australians | 500 | 108 | MINI | PHQ-9 |
| Ghazisaeedi | 2021 | Int J Ment Health Addict | 3.8 | Iran | Undergraduate medical students | 400 | NR | SCID | PHQ-2; PHQ-9; WHO-5 |
| Gholizadeh | 2019 | Contemp Nurse | 1.8 | Iran | Coronary artery disease inpatients aged 20 or above | 150 | 33 | MINI | PHQ-9 |
| Gouweloos-Trines | 2019 | Eur J Psychotraumatol | 4.1 | Netherlands | Survivors of an airplane crash aged 14 years or older | 38 | 6 | MINI | PHQ-2 |
| Green | 2018 | J Affect Disord | 4.8 | Kenya | Perinatal women (pregnant and postpartum) | 192 | 10 | SCID-5 | PDEPS; EPDS; PHQ-9 |
| Guerin | 2018 | J Affect Disord | 4.8 | United States | Adults aged 18 to 54 versus 55 to 80 | 311 | 117 | SCID-IV or DIGS | GDS-15 |
| Hamers | 2018 | J Appl Res Intellect Disabil | 2.7 | Netherlands | People with intellectual disabilities aged 18 to 49 years | 43 | 8 | PAS‐ADD | ADAMS- Depressive Mood Subscale |
| Herizchi | 2020 | Health Promot Perspect | 0.6 | Iran | Adults 60 years and older | 387 | 44 | SCID-IV | GDS |
| Hirt | 2020 | J Affect Disord | 4.8 | Netherlands;  Germany | Hospitalized patients with a diagnosis of ischemic stroke or intracerebral hemorrhage | 93 | 17 | CIDI | DePreS |
| Hitchon | 2020 | Arthritis Care Res | 4.8 | Canada | Outpatients with rheumatoid arthritis aged 18 or older | 150 | 17 | SCID-IV | PHQ-2; PHQ-9; HADS-D |
| Housen | 2018 | Transcult Psychiatry | 2.2 | India | Adults general medical outpatients aged 18 years or older | 290 | 81 | MINI | HSCL-Depression subscale |
| Indu | 2018 | Asian J Psychiatr | 3.5 | India | Women aged 18-60 years in primary care | 228 | 57 | MINI | PHQ-9 |
| Jahn | 2018 | Psychiatr Prax | 1.5 | Austria | Adults from the general population | 508 | 37 | SCAN | CES-D |
| Jokelainen | 2019 | Scand J Prim Health Care | 2.6 | Finland | Older adults born in 1935 | 505 | 24 | MINI | SDS; BDI-21 |
| Kagee | 2020 | Gen Hosp Psychiatry | 3.2 | South Africa | Patients receiving outpatient HIV care | 688 | 170 | SCID-5 | CESD-R |
| Karam | 2018 | Alzheimers Dement (Amst) | 21.6 | Lebanon | Individuals aged between 63 and 101 years | 56 | 10 | SCID | CSDD; GDS; HADS-D |
| Kim | 2020 | Seizure | 3.2 | Korea | Outpatients aged 18 or older with epilepsy | 213 | 48 | MINI | NDDI-E; PHQ-9 |
| Kokoszka | 2020 | Prim Care Diabetes | 2.5 | Poland | Outpatients and inpatients with Type 2 diabetes aged 18 or older | 101 | 35 | MINI | BDI; HADS-D; DDS-RS; BSSDA – depression |
| Kwan | 2019 | Semin Arthritis Rheum | 5.5 | Canada | Adult outpatients with systemic lupus erythematosus | 159 | 23 | MINI | CES-D; HADS-D |
| Kyranou | 2020 | BMC Psychiatry | 3.6 | Greece | Outpatients receiving chemotherapy for solid tumours | 152 | NR | MINI | DT |
| Lafont | 2021 | Oncologist | 5 | France | Inpatients and outpatients aged 70 or older with solid or hematological cancers | 830 | 208 | Clinician interview | GDS-4 |
| Legha | 2020 | Confl Health | 2.7 | Haiti | Transitional age youth (18-22) attending schools | 120 | 9 | SCID-IV | ZLDSI |
| Liu | 2019 | Neuropsychiatr Dis Treat | 2.6 | Taiwan | Outpatients with chronic low back pain | 225 | 21 | SCID-IV | DSSS-DS |
| Loades | 2020 | Eur Child Adolesc Psychiatry | 4.8 | England | Adolescents (12-18 years) with Chronic Fatigue Syndrome/Myalgic Encephalomyelitis | 164 | 33 | K-SADS | RCADS; HADS-D |
| Lydsdottir | 2019 | Midwifery | 2.4 | Iceland | Pregnant women | 474 | NR | MINI-Plus | EPDS |
| Macêdo | 2018 | Lupus | 2.9 | Brazil | Patients with systemic lupus erythematosus | 108 | 21 | DSM-5 diagnosis by clinician | BDI ; CES-D ; HADS-D |
| Marrie | 2018 | Mult Scler Relat Disord | 4.3 | Canada | Outpatients with multiple sclerosis aged 18 years or older, attending a multiple sclerosis clinic | 253 | 26 | SCID-IV | PHQ-2; PHQ-9; HADS-D; PROMIS Depression; K6 |
| Martínez | 2020 | J Clin Psychol | 2.9 | Chile | Mothers 18 years and older with infants aged 2-6 months | 298 | 63 | MINI | EPDS-3; EPDS |
| Massai | 2018 | Parkinsons Dis | 2.7 | Italy | Patients with Parkinson’s disease | 74 | 23 | DSM-5 diagnosis by physician | GDS |
| Matthey | 2019 | J Affect Disord | 4.8 | Australia | Pregnant women attending antenatal clinic | 247 | 7 | MINI | MGMQ; EPDS |
| McCartney | 2020 | Epilepsy Behav | 2.9 | Australia | Adult inpatients aged 18 to 77 years with epilepsy or psychogenic non-epileptic seizure | 485 | 115 | DSM-IV or DSM-5 diagnosis by neuropsychiatry team | HADS-D |
| Mohsin | 2021 | Int J Environ Res Public Health | 3.4 | Pakistan | Pregnant women and mothers with children aged 3 years or less | 425 | 100 | SCID-IV | CIDT-MD |
| Molebatsi | 2020 | BMC Psychiatry | 3.6 | Botswana | Adult primary care patients | 257 | 105 | MINI | PHQ-9 |
| Muramatsu | 2018 | Gen Hosp Psychiatry | 3.2 | Japan | Primary care patients | 284 | 93 | MINI-Plus | PHQ-9 |
| Nabbe | 2019 | PloS One | 3.2 | France | General practice patients aged 18 or older | 142 | 73 | PSE-9 | HSCL-25 |
| Navarrete | 2019 | Salud Publica Mex | 2 | Mexico | Women during pregnancy | 210 | NR | SCID-IV | Whooley; Arroll question |
| Park | 2020 | Front Psychol | 3 | Korea | Adults recruited online and during visit to hospital (patients, visitors, staff) | 1145 | 96 | MINI-Plus | BDI-II |
| Peng | 2020 | Dermatol Ther | 2.9 | China | Outpatients with acne aged 18 to 24 years | 258 | 47 | SCID | PHQ-9 |
| Rancans | 2018 | Ann Gen Psychiatry | 3.5 | Latvia | Primary care outpatients aged 18 years or older | 1467 | 150 | MINI | PHQ-9; PHQ-2 |
| Rashid | 2019 | Epilepsy Behav | 2.9 | India | Outpatients with epilepsy aged 18 or older | 217 | 90 | MINI | NDDI-E |
| Rashid | 2021 | Epilepsy Behav | 2.9 | India | Adult outpatients with epilepsy | 449 | 180 | MINI | NDDI-E; PHQ-9 |
| Recklitis | 2020 | Cancer | 6.9 | United States | Adult cancer survivors aged 18 to 40 years | 249 | 32 | SCID-IV | PROMIS-D-SF |
| Risal | 2019 | J Nepal Health Res Counc | NA | Nepal | Adult outpatients aged 60 years or older | 106 | 51 | ICD-10 diagnosis by psychiatrist | GDS-15 |
| Rodríguez-Mayoral | 2018 | Palliat Support Care | 2.3 | Mexico | Outpatients aged 18 and older with advanced cancer receiving palliative care | 70 | 14 | DSM-5 diagnosis by psychiatrist | BEDS |
| Saal | 2018 | AIDS Care | 2.3 | South Africa | Individuals aged 18 years or older seeking HIV testing | 500 | 72 | SCID-RV | BDI-I |
| Saldivia | 2019 | Rev Med Chil | 0.6 | Chile | Primary care patients aged 18 to 75 years | 1738 | 187 | CIDI | PHQ-9 |
| Sampasa-Kanyinga | 2018 | PloS One | 3.2 | Canada | Military personnel | 6700 | 208 | CIDI | K10 |
| Sasaki | 2019 | J Obstet Gynaecol Res | 1.7 | Japan | Women 4 days to 1 month postpartum | 80 | 9 | DSM-5 diagnosis by psychiatrist | EPDS; CES-D |
| Scoppetta | 2021 | J Affect Disord | 4.8 | Colombia | Adult primary care patients | 243 | 52 | MINI | PHQ-2 |
| Searle | 2019 | Assessment | 4.7 | Australia | Regular-serving military personnel | 1730 | 74 | CIDI | K10; PHQ-9 |
| Shaheen | 2019 | Am J Mens Health | 2.8 | Saudi Arabia | Fathers of newborns aged up to 6 months | 57 | 9 | DSM-5 diagnosis by psychologist | EPDS |
| Shih | 2020 | Seizure | 3.2 | Taiwan | Inpatients and outpatients with epilepsy aged 20 or older | 109 | 20 | MINI | NDDI-E |
| Silagadze | 2019 | Epilepsy Behav | 2.9 | Georgia | Adult outpatients with epilepsy | 130 | 31 | ICD-10 diagnosis by psychiatrist | NDDI-E |
| Smith Fawzi | 2019 | Neurol Psychiatry Brain Res | NA | Tanzania | Primary care patients aged 18 years and older | 174 | 18 | MINI | PHQ-9 |
| Smith-Nielsen | 2018 | BMC Psychiatry | 3.6 | Denmark | Women aged 18 or older who were 2 to 10 months postpartum | 324 | 118 | SCID-5; ICD-10 diagnosis by clinical psychologist | EPDS |
| Tan | 2018 | J Am Psychiatr Nurses Assoc | 2.4 | Singapore | Adult outpatients aged 65 years or older attending a general geriatric outpatient clinic | 77 | 6 | DSM-IV diagnosis by geriatrician | Emoticon scale |
| Tran | 2019 | J Affect Disord | 4.8 | Indonesia | Adolescents (aged between 16 and 18 years) attending senior schools | 189 | NR | MINI KID | CESD-R 60, CESD-R 80, K10, K6 |
| Tschorn | 2019 | Psychiatr Prax | 1.5 | Germany | Patients with coronary heart disease | 1019 | 83 | CIDI | PHQ-9; HADS-D |
| Udedi | 2019 | BCM Psychiatry | 3.6 | Malawi | Outpatients with type-2 diabetes | 323 | 58 | SCID-IV | PHQ-9 |
| Urtasun | 2019 | BMC Psychiatry | 3.6 | Argentina | Adults attending primary care or mental health clinics | 169 | 102 | MINI | PHQ-9 |
| van Heyningen | 2018 | Plos One | 3.5 | South Africa | Pregnant women attending a primary care antenatal clinic | 376 | 81 | MINI Plus | EPDS; PHQ-9; K10; Whooley; EPDS-3; K6; PHQ-2; Whooley + help |
| van Heyningen | 2019 | Glob Ment Health | 3.2 | South Africa | Pregnant women aged 18 years or older receiving outpatient antenatal care | 376 | 81 | MINI | Whooley; EPDS; PHQ-9 |
| Vázquez | 2019 | J Affect Disord | 4.8 | Spain | Pregnant women | 569 | 40 | SCID-IV | EPDS |
| Vogeli | 2018 | Res Nurs Health | 2.2 | United States | Mothers of 4- to 15-month-old infants | 238 | 36 | SCID-IV | PDSS |
| Vrublevska | 2018 | Nord J Psychiatry | 2.2 | Latvia | Adult (aged 18 years or older) primary care patients | 272 | 37 | MINI | PHQ-9 |
| Wang | 2018 | J Geriatr Psychiatry Neurol | 1.8 | United States | Older adult outpatients with ischemic heart disease and prior stroke | 147 | 35 | DIS | CES-D ; PHQ-9; PHQ-2 ; Whooley |
| Wang | 2019 | Ther Apher Dial | 2.7 | China | Patients aged 18 years or older undergoing maintenance hemodialysis | 112 | 24 | SCID-I | DI-MHD; BDI |
| Weihs | 2018 | Psychooncology | 3.9 | United States | Patients with breast cancer | 82 | 14 | CIDI | PHQ-4; DRQ-7 |
| Williams | 2020 | Neurol Clin Pract | 4.7 | Canada | Outpatients with neurologic disorders aged 18 or older | 830 | NR | SCID-IV | PHQ-9 |
| Williams | 2021 | Assessment | NA | United States | Adults with autism spectrum disorder aged 18 to 45 years | 66 | 24 | SCID-5; MINI | BDI-II |
| Woldetensay | 2018 | PloS One | 3.2 | Ethiopia | Pregnant women | 246 | 28 | MINI-Plus | PHQ-9 |
| Xia | 2019 | Epilepsy Behav | 2.9 | China | Adults outpatients with epilepsy | 213 | 35 | MINI | PHQ-9; PHQ-2 |
| Xia | 2020 | Epilepsy Behav | 2.9 | China | Patients with epilepsy aged 18 and older | 213 | 35 | MINI | NDDI-E; HADS-D |
| Yang | 2018 | Psychol Asses | 5.1 | China | Adolescents (grades 7-12) | 612 | 44 | K-SADS | CES-D |
| Yardeni | 2020 | Psychooncology | 3.9 | Israel | Patients with cancer aged 7 to 21 years | 91 | 11 | K-SADS | PROMIS Depression module |
| Ye | 2020 | BMJ Open | 2.7 | China | Outpatients and inpatients with psoriasis | 148 | 30 | DSM-5 diagnosis by physician | PHQ-9 |
| Yuan | 2019 | J Psychosom Res | 3 | China | Inpatients with acute coronary syndrome | 782 | 122 | MINI | PHQ-9; HADS-D |
| Zachar-Tirado | 2021 | J Head Trauma Rehabil | 2.7 | United States | Outpatients with traumatic brain injury aged 12 to 17 years | 101 | 42 | DSM-5 diagnosis by a neuropsychologist | PHQ-A; PHQ-A_2 |
| Zinchuk | 2020 | Epilepsy Behav | 2.9 | Russia | Adult inpatients and outpatients with epilepsy | 175 | 76 | MINI | NDDI-E |

Abbreviations: ADAMS = Anxiety, Depression And Mood Scale; AviDI-18 = Akena Visual Depression Inventory; APC = Adult Primary Care; BDI = Beck Depression Inventory; BDI-I = Beck Depression Inventory-I; BDI-II = Beck Depression Inventory-II; BEDS = Brief Edinburgh Depression Scale; BSSDA = Brief Self-Rating Scale of Depression and Anxiety; CD-RISC 10 = Connor-Davidson Resilience Scale-10; CDI = Cognitive Depression Index; CDST = Children Depression Screening Tool; CES-D = Center for Epidemiologic Studies Depression Scale; CESD-R = Center for Epidemiological Studies Depression Scale Revised; CESD-R 60 = Center for Epidemiological Studies Depression Scale Revised 60; CESD-R 80 = Center for Epidemiological Studies Depression Scale Revised 80; CIDI = Composite International Diagnostic Interview; CIDT-MD = Community Informant Detection Tool for Maternal Depression; CSDD = Cornell Scale for Depression in Dementia; DDS-RS = Depression in Diabetes Self-Rating Scale; DEPQ = Depression Question; DePreS = Post-stroke Depression Prediction Scale; DI-MHD = Depression Inventory for Maintenance Hemodialysis Patients; DIGS = Diagnostic Interview for Genetic Studies; DIS = Diagnostic Interview Schedule; DRQ-7 = Depression Risk Questionnaire 7; DSM-5 = Diagnostic and Statistical Manual of Mental Disorders, 5^th^ Edition; DSM-IV = Diagnostic and Statistical Manual of Mental Disorders, fourth edition; DSSS-DS = Depression and Somatic Symptoms Scale – Depression Subscale (DS); DT = Distress Thermometer; EPDS = Edinburgh Postnatal Depression Scale; EPDS-3 = Edinburgh Postnatal Depression Scale-3; GDS = Geriatric Depression Scale; GDS-15 = Geriatric Depression Scale-15; GDS-30 = Geriatric Depression Scale-30; GDS-4 = Geriatric Depression Scale-4; GDS-5 = Geriatric Depression Scale-5; HADS-D = Hospital Anxiety and Depression Scale – Depression subscale; HSCL = Hopkins Symptom Checklist; HSCL-25 = Hopkins Symptom Checklist-25; ICD-10 = International Classification of Diseases, Tenth; K-SADS = Kiddie Schedule for Affective Disorders and Schizophrenia; K-SADS-PL = Schedule for Affective Disorders and Schizophrenia for School-Age Children – Present and Lifetime Version; K10 = Kessler Psychological Distress Scale-10; K6 = Kessler Psychological Distress Scale-6; MFQ = Mood and Feeling Questionnaire; MGMQ = Matthey Generic Mood Questionnaire; MINI = Mini-International Neuropsychiatric Interview; MINI KID = Mini International Neuropsychiatric Interview for Children and Adolescents; MINI Plus = Mini-International Neuropsychiatric Interview-Plus; NDDI-E = Neurologic Depression Disorders Inventory in Epilepsy; NR = Not Reported; PAS‐ADD = Psychiatric Assessment Schedule for Adults with Developmental Disability; PDEPS = Perinatal Depression Screening; PDPI-R = Postpartum Depression Predictors Inventory-Revised; PDSS = Postpartum Depression Screening Scale; PHQ-2 = Patient Health Questionnaire-2; PHQ-4 = Patient Health Questionnaire-4; PHQ-9 = Patient Health Questionnaire-9; PHQ-A = Patient Health Questionnaire – Adolescent; PHQ-A_2 = Patient Health Questionnaire – Adolescent (2-item version); PROMIS = Patient Reported Outcomes Measurement Information System; PROMIS-D-CAT = Patient Reported Outcomes Measurement Information System Depression Computer Adaptive Test; PROMIS-D-SF = Patient Reported Outcomes Measurement Information System Depression Short Form; RCADS = Revised Children’s Anxiety and Depression Scale; PSE-9 = Present State Examination-9; SCAN = Schedules for Clinical Assessment in Neuropsychiatry; SCID = Structured Clinical Interview for DSM; SCID-RV = Structured Clinical Interview for the DSM-Research Version; SCID-5 = Structured Clinical Interview for DSM-5; SCID-I = Structured Clinical Interview for DSM-IV Axis I Disorders; SCID-IV = Structured Clinical Interview for DSM-IV; SDS = Self-Rating Depression Scale; WHO-5 = World Health Organization Wellbeing Index; WMH-ICS = World Mental Health-International College Student; ZLDSI = Zanmi Lasante Depression Symptom Inventory.

**Appendix 3. Results by Study for Adherence with STARD**

| **First Author Last Name** | **Year** | **Journal** | **Number of STARD Items that were Adequately Reported** | **Number of STARD Items that were Partially Reported** | **Number of STARD Items that were Inadequately or Not reported** | **Number of STARD Items that were Not Applicable** |
| --- | --- | --- | --- | --- | --- | --- |
| Akena | 2018 | Br J Psychiatry | 9 | 10 | 11 | 4 |
| Alves | 2019 | Midwifery | 11 | 6 | 13 | 4 |
| Arturo Cassiani-Miranda | 2021 | Gen Hosp Psychiatry | 9 | 14 | 7 | 4 |
| Aslan | 2020 | Front Psychiatry | 13 | 8 | 9 | 4 |
| Baldellou Lopez | 2021 | J Psychosom Res | 13 | 7 | 10 | 4 |
| Ballester | 2019 | PLoS One | 13 | 8 | 9 | 4 |
| Baumgartner | 2019 | Psychiatr Prax | 9 | 9 | 12 | 4 |
| Bautovich | 2018 | Australas Psychiatry | 9 | 9 | 12 | 4 |
| Bernstein | 2018 | Inflamm Bowel Dis | 10 | 10 | 10 | 4 |
| Bhana | 2019 | S Afr Med J | 12 | 9 | 9 | 4 |
| Binagwaho | 2021 | BMC Pediatr | 9 | 10 | 11 | 4 |
| Blanco | 2019 | Aging Ment Health | 11 | 11 | 9 | 3 |
| Borghero | 2018 | Rev Med Chil | 12 | 8 | 10 | 4 |
| Butnoriene | 2018 | BMC Psychiatry | 16 | 5 | 9 | 4 |
| Cassiani-Miranda | 2021 | Rev Colomb Psiquiatr | 12 | 9 | 9 | 4 |
| Chenneville | 2019 | J Affect Disord | 9 | 4 | 17 | 4 |
| Clover | 2018 | Qual Life Res | 16 | 8 | 6 | 4 |
| Colomo | 2021 | Arch Bronconeumol | 10 | 8 | 12 | 4 |
| Cruzado | 2018 | Support Care Cancer | 10 | 9 | 11 | 4 |
| Cumbe | 2020 | BMC Psychiatry | 10 | 8 | 13 | 3 |
| Dajpratham | 2020 | BMC Psychiatry | 18 | 4 | 8 | 4 |
| Degefa | 2020 | BMC Psychiatry | 13 | 12 | 6 | 3 |
| Durmaz | 2018 | North Clin Istanb | 9 | 9 | 12 | 4 |
| Eriksen | 2019 | J Affect Disord | 13 | 8 | 9 | 4 |
| Figueiredo-Duarte | 2019 | Aging Ment Health | 8 | 10 | 13 | 3 |
| Fuseekul | 2021 | Child Adolesc Psychiatry Ment Health | 13 | 12 | 5 | 4 |
| Gallis | 2018 | PeerJ | 16 | 10 | 4 | 4 |
| Getting it Right Collaborative Group | 2019 | Med J Aust | 16 | 5 | 9 | 4 |
| Ghazisaeedi | 2021 | Int J Ment Health Addict | 8 | 8 | 14 | 4 |
| Gholizadeh | 2019 | Contemp Nurse | 15 | 9 | 6 | 4 |
| Gouweloos-Trines | 2019 | Eur J Psychotraumatol | 11 | 13 | 7 | 3 |
| Green | 2018 | J Affect Disord | 15 | 8 | 7 | 4 |
| Guerin | 2018 | J Affect Disord | 13 | 7 | 10 | 4 |
| Hamers | 2018 | J Appl Res Intellect Disabil | 9 | 10 | 12 | 3 |
| Herizchi | 2020 | Health Promot Perspect | 9 | 8 | 13 | 4 |
| Hirt | 2020 | J Affect Disord | 14 | 13 | 3 | 4 |
| Hitchon | 2020 | Arthritis Care Res | 10 | 8 | 12 | 4 |
| Housen | 2018 | Transcult Psychiatry | 13 | 9 | 9 | 3 |
| Indu | 2018 | Asian J Psychiatr | 12 | 5 | 13 | 4 |
| Jahn | 2018 | Psychiatr Prax | 12 | 10 | 8 | 4 |
| Jokelainen | 2019 | Scand J Prim Health Care | 15 | 7 | 8 | 4 |
| Kagee | 2020 | Gen Hosp Psychiatry | 11 | 9 | 10 | 4 |
| Karam | 2018 | Alzheimers Dement (Amst) | 10 | 12 | 8 | 4 |
| Kim | 2020 | Seizure | 12 | 7 | 11 | 4 |
| Kokoszka | 2020 | Prim Care Diabetes | 12 | 8 | 10 | 4 |
| Kwan | 2019 | Semin Arthritis Rheum | 14 | 5 | 11 | 4 |
| Kyranou | 2020 | BMC Psychiatry | 7 | 12 | 11 | 4 |
| Lafont | 2021 | Oncologist | 12 | 10 | 9 | 3 |
| Legha | 2020 | Confl Health | 13 | 8 | 9 | 4 |
| Liu | 2019 | Neuropsychiatr Dis Treat | 10 | 9 | 11 | 4 |
| Loades | 2020 | Eur Child Adolesc Psychiatry | 9 | 12 | 9 | 4 |
| Lydsdottir | 2019 | Midwifery | 16 | 6 | 8 | 4 |
| Macêdo | 2018 | Lupus | 13 | 7 | 10 | 4 |
| Marrie | 2018 | Mult Scler Relat Disord | 14 | 9 | 7 | 4 |
| Martínez | 2020 | J Clin Psychol | 13 | 7 | 10 | 4 |
| Massai | 2018 | Parkinsons Dis | 9 | 9 | 12 | 4 |
| Matthey | 2019 | J Affect Disord | 9 | 12 | 9 | 4 |
| McCartney | 2020 | Epilepsy Behav | 10 | 9 | 11 | 4 |
| Mohsin | 2021 | Int J Environ Res Public Health | 10 | 7 | 13 | 4 |
| Molebatsi | 2020 | BMC Psychiatry | 15 | 8 | 7 | 4 |
| Muramatsu | 2018 | Gen Hosp Psychiatry | 9 | 10 | 11 | 4 |
| Nabbe | 2019 | PLoS One | 11 | 7 | 12 | 4 |
| Navarrete | 2019 | Salud Publica Mex | 13 | 8 | 9 | 4 |
| Park | 2020 | Front Psychol | 11 | 9 | 10 | 4 |
| Peng | 2020 | Dermatol Ther | 9 | 9 | 12 | 4 |
| Rancans | 2018 | Ann Gen Psychiatry | 12 | 8 | 10 | 4 |
| Rashid | 2019 | Epilepsy Behav | 8 | 7 | 15 | 4 |
| Rashid | 2021 | Epilepsy Behav | 7 | 10 | 13 | 4 |
| Recklitis | 2020 | Cancer | 15 | 9 | 8 | 2 |
| Risal | 2019 | J Nepal Health Res Counc | 9 | 12 | 10 | 3 |
| Rodríguez-Mayoral | 2018 | Palliat Support Care | 12 | 8 | 10 | 4 |
| Saal | 2018 | AIDS Care | 7 | 14 | 9 | 4 |
| Saldivia | 2019 | Rev Med Chil | 8 | 11 | 11 | 4 |
| Sampasa-Kanyinga | 2018 | PLoS One | 12 | 7 | 11 | 4 |
| Sasaki | 2019 | J Obstet Gynaecol Res | 12 | 7 | 11 | 4 |
| Scoppetta | 2021 | J Affect Disord | 10 | 13 | 7 | 4 |
| Searle | 2019 | Assessment | 17 | 8 | 5 | 4 |
| Shaheen | 2019 | Am J Mens Health | 11 | 8 | 11 | 4 |
| Shih | 2020 | Seizure | 8 | 10 | 12 | 4 |
| Silagadze | 2019 | Epilepsy Behav | 8 | 10 | 12 | 4 |
| Smith Fawzi | 2019 | Neurol Psychiatry Brain Res | 8 | 13 | 9 | 4 |
| Smith-Nielsen | 2018 | BMC Psychiatry | 14 | 6 | 10 | 4 |
| Tan | 2018 | J Am Psychiatr Nurses Assoc | 16 | 6 | 8 | 4 |
| Tran | 2019 | J Affect Disord | 11 | 8 | 11 | 4 |
| Tschorn | 2019 | Psychiatr Prax | 12 | 6 | 12 | 4 |
| Udedi | 2019 | BCM Psychiatry | 14 | 8 | 9 | 3 |
| Urtasun | 2019 | BMC Psychiatry | 12 | 10 | 8 | 4 |
| van Heyningen | 2018 | PLos One | 12 | 7 | 12 | 3 |
| van Heyningen | 2019 | Glob Ment Health | 12 | 6 | 12 | 4 |
| Vázquez | 2019 | J Affect Disord | 11 | 9 | 10 | 4 |
| Vogeli | 2018 | Res Nurs Health | 9 | 8 | 13 | 4 |
| Vrublevska | 2018 | Nord J Psychiatry | 16 | 8 | 6 | 4 |
| Wang | 2018 | J Geriatr Psychiatry Neurol | 13 | 10 | 7 | 4 |
| Wang | 2019 | Ther Apher Dial | 12 | 7 | 11 | 4 |
| Weihs | 2018 | Psychooncology | 14 | 6 | 11 | 3 |
| Williams | 2020 | Neurol Clin Pract | 14 | 8 | 8 | 4 |
| Williams | 2021 | Assessment | 9 | 8 | 13 | 4 |
| Woldetensay | 2018 | PLoS One | 12 | 7 | 11 | 4 |
| Xia | 2019 | Epilepsy Behav | 10 | 7 | 14 | 3 |
| Xia | 2020 | Epilepsy Behav | 9 | 8 | 14 | 3 |
| Yang | 2018 | Psychol Asses | 10 | 8 | 12 | 4 |
| Yardeni | 2020 | Psychooncology | 10 | 8 | 12 | 4 |
| Ye | 2020 | BMJ Open | 7 | 7 | 16 | 4 |
| Yuan | 2019 | J Psychosom Res | 11 | 9 | 10 | 4 |
| Zachar-Tirado | 2021 | J Head Trauma Rehabil | 13 | 8 | 10 | 3 |
| Zinchuk | 2020 | Epilepsy Behav | 10 | 9 | 11 | 4 |

**Appendix 4. Results by Study and Item for Adherence with STARD**

|  |  | **STARD Item Number** | | | | | | | | | | | | | | | | | | | | | | | | | | | | | | | | | |
| --- | --- | --- | --- | --- | --- | --- | --- | --- | --- | --- | --- | --- | --- | --- | --- | --- | --- | --- | --- | --- | --- | --- | --- | --- | --- | --- | --- | --- | --- | --- | --- | --- | --- | --- | --- |
| **First Author Last Name** | **Year** | **1** | **2** | **3** | **4** | **5** | **6** | **7** | **8** | **9** | **10a** | **10b** | **11** | **12a** | **12b** | **13a** | **13b** | **14** | **15** | **16** | **17** | **18** | **19** | **20** | **21a** | **21b** | **22** | **23** | **24** | **25** | **26** | **27** | **28** | **29** | **30** |
| Akena | 2018 | A | P | A | A | A | I | I | P | I | P | P | I | I | N | N | P | A | N | I | P | A | I | A | P | P | I | P | I | N | A | A | I | I | P |
| Alves | 2019 | A | P | A | P | A | A | I | A | I | A | A | I | I | N | N | I | A | N | I | I | I | I | A | I | I | A | P | P | N | P | A | I | I | P |
| Arturo Cassiani-Miranda | 2021 | A | P | A | P | A | A | P | P | I | P | P | P | I | N | N | P | A | N | I | I | A | I | P | P | P | A | P | P | N | A | A | I | I | P |
| Aslan | 2020 | A | P | A | P | A | A | A | A | I | A | A | P | I | N | N | A | A | N | I | I | I | I | P | P | P | A | P | I | N | P | A | I | I | A |
| Baldellou Lopez | 2021 | A | P | A | A | A | A | I | I | I | P | P | A | A | N | N | I | A | N | I | I | I | I | A | P | P | A | P | P | N | A | A | I | I | A |
| Ballester | 2019 | A | P | A | P | A | A | A | A | A | A | A | P | I | N | N | A | A | N | P | I | I | P | A | I | I | I | P | I | N | P | A | I | I | P |
| Baumgartner | 2019 | A | P | A | P | A | A | I | P | A | P | P | P | I | N | N | A | A | N | I | P | I | I | A | I | I | I | P | I | N | P | A | I | I | I |
| Bautovich | 2018 | A | P | A | P | A | A | I | P | I | P | P | I | I | N | N | P | A | N | I | I | I | I | A | A | A | I | P | I | N | P | A | I | I | P |
| Bernstein | 2018 | A | P | P | P | A | A | A | P | I | P | P | I | I | N | N | P | A | N | I | I | A | I | A | I | I | A | P | P | N | P | A | I | I | A |
| Bhana | 2019 | A | P | A | P | A | A | A | P | A | P | A | I | I | N | N | P | A | N | I | I | A | I | P | I | I | A | P | P | N | P | A | I | I | A |
| Binagwaho | 2021 | A | P | P | P | A | P | I | A | A | P | P | P | A | N | N | I | A | N | I | I | A | I | A | I | I | I | P | I | N | P | A | I | I | P |
| Blanco | 2019 | A | P | A | P | A | A | I | P | A | P | P | I | I | N | A | A | A | N | P | I | I | I | A | P | P | A | P | I | N | P | A | I | I | P |
| Borghero | 2018 | A | P | A | P | A | A | A | A | A | P | P | I | I | N | N | I | P | N | I | I | A | I | A | A | A | I | P | I | N | P | A | I | I | P |
| Butnoriene | 2018 | A | P | A | A | A | P | A | A | A | P | A | A | A | N | N | I | A | N | I | A | I | I | A | I | I | I | P | A | N | P | A | I | I | A |
| Cassiani-Miranda | 2021 | A | P | A | P | A | A | A | P | I | P | A | A | A | N | N | I | A | N | I | I | P | I | P | I | I | A | P | P | N | A | A | I | I | P |
| Chenneville | 2019 | A | P | A | P | A | I | I | A | I | P | I | I | I | N | N | I | A | N | I | I | I | I | A | I | I | I | P | I | N | A | A | I | I | A |
| Clover | 2018 | A | P | A | A | A | A | P | P | A | A | P | I | P | N | N | P | A | N | I | P | A | I | A | A | A | I | A | A | N | A | A | I | I | P |
| Colomo | 2021 | A | A | A | P | A | A | A | A | I | P | P | I | I | N | N | P | A | N | I | I | I | I | A | P | P | I | P | I | N | P | A | I | I | I |
| Cruzado | 2018 | A | P | A | P | A | A | A | P | I | P | P | I | A | N | N | I | A | N | I | I | I | I | A | P | P | I | P | A | N | P | A | I | I | I |
| Cumbe | 2020 | A | A | A | P | A | P | I | A | A | P | P | I | I | N | I | P | A | N | I | I | I | I | A | I | I | I | P | P | N | P | A | I | I | A |
| Dajpratham | 2020 | A | A | A | P | A | A | A | A | I | P | A | I | A | N | N | A | A | N | I | I | I | I | A | A | A | A | P | P | N | A | A | I | I | A |
| Degefa | 2020 | A | A | P | P | A | A | A | A | A | P | P | I | I | N | P | P | A | N | P | P | A | I | A | P | P | A | P | I | N | P | A | I | I | A |
| Durmaz | 2018 | A | A | A | P | A | A | I | P | I | P | P | I | I | N | N | I | A | N | I | I | P | I | A | P | P | I | P | I | N | P | A | I | I | A |
| Eriksen | 2019 | A | P | A | P | A | A | I | P | I | P | P | I | I | N | N | P | A | N | A | I | I | I | A | A | A | A | P | P | N | A | A | I | I | A |
| Figueiredo-Duarte | 2019 | A | P | A | P | A | A | P | P | I | P | I | I | I | N | I | I | A | N | I | I | A | I | P | P | P | I | P | I | N | P | A | I | I | A |
| Fuseekul | 2021 | A | P | P | P | A | P | A | P | I | A | A | A | I | N | N | P | A | N | A | I | P | P | P | A | A | A | P | P | N | P | A | I | I | A |
| Gallis | 2018 | A | P | A | P | A | A | A | A | A | P | A | P | A | N | N | P | A | N | A | I | P | I | P | A | A | A | P | P | N | P | A | I | I | A |
| Getting it Right Collaborative Group | 2019 | A | A | A | P | A | A | I | A | A | A | P | I | I | N | N | A | A | N | A | I | A | A | A | I | I | A | P | P | N | I | A | I | I | P |
| Ghazisaeedi | 2021 | A | P | A | P | A | I | I | P | I | A | P | I | A | N | N | I | A | N | I | I | I | I | A | P | P | I | P | I | N | P | A | I | I | I |
| Gholizadeh | 2019 | A | P | A | P | A | A | A | A | A | A | A | P | A | N | N | P | A | N | I | I | A | I | A | P | P | A | P | I | N | P | A | I | I | P |
| Gouweloos-Trines | 2019 | A | P | A | P | A | P | P | I | I | P | P | A | I | N | A | A | A | N | P | I | I | P | A | P | P | A | P | P | N | A | A | I | I | P |
| Green | 2018 | A | P | A | P | A | A | I | P | A | A | P | I | I | N | N | A | A | N | P | P | I | A | A | A | A | A | P | I | N | A | A | I | I | P |
| Guerin | 2018 | A | P | A | A | A | P | I | I | I | A | P | I | A | N | N | A | A | N | I | P | I | I | A | A | A | I | P | P | N | P | A | I | I | A |
| Hamers | 2018 | A | P | A | P | A | A | A | P | I | P | P | I | I | N | I | I | A | N | I | I | I | I | A | P | P | I | P | P | N | A | A | I | I | P |
| Herizchi | 2020 | A | P | A | P | A | A | I | A | A | P | P | P | I | N | N | I | A | N | I | I | A | I | P | I | I | I | P | I | N | I | A | I | I | P |
| Hirt | 2020 | A | A | A | P | A | P | P | A | A | P | P | P | A | N | N | P | A | N | I | I | A | P | A | P | P | A | P | P | N | P | A | A | I | A |
| Hitchon | 2020 | A | P | A | P | A | I | I | A | I | A | P | I | I | N | N | P | A | N | A | I | I | I | A | I | I | A | P | P | N | P | A | I | I | P |
| Housen | 2018 | I | P | P | P | A | A | I | P | A | A | A | A | A | N | P | P | A | N | I | I | I | I | A | A | A | I | P | P | N | P | A | I | I | A |
| Indu | 2018 | A | A | A | P | A | A | I | A | I | P | P | I | I | N | N | I | A | N | I | I | A | A | A | I | I | A | P | P | N | I | A | I | I | I |
| Jahn | 2018 | A | P | A | P | A | P | I | P | A | P | P | P | A | N | N | A | A | N | I | P | I | I | A | A | A | A | P | I | N | P | A | I | I | I |
| Jokelainen | 2019 | A | P | A | P | A | A | A | A | A | P | P | I | A | N | N | A | A | N | I | I | I | I | A | A | A | A | P | P | N | P | A | I | I | I |
| Kagee | 2020 | A | A | A | P | A | A | A | P | I | A | A | P | I | N | N | I | A | N | I | I | I | I | A | P | P | I | P | P | N | P | A | I | I | P |
| Karam | 2018 | A | P | A | P | A | P | P | P | A | P | P | I | A | N | N | P | A | N | A | I | I | I | A | P | P | I | P | I | N | A | A | I | I | P |
| Kim | 2020 | A | P | A | P | A | A | P | A | I | P | A | P | I | N | N | I | A | N | I | I | I | I | A | A | A | I | P | I | N | P | A | I | I | A |
| Kokoszka | 2020 | A | P | A | P | A | A | A | P | I | P | P | I | I | N | N | I | A | N | I | I | A | I | A | A | A | I | P | P | N | P | A | I | I | A |
| Kwan | 2019 | A | P | A | P | A | A | I | A | A | P | A | A | I | N | N | I | A | N | I | P | I | I | A | I | I | A | P | I | N | A | A | I | I | A |
| Kyranou | 2020 | A | P | A | P | A | I | I | P | I | P | P | P | I | N | N | P | A | N | I | I | I | I | A | P | P | I | P | P | N | P | A | I | I | A |
| Lafont | 2021 | A | P | A | P | A | I | I | A | A | P | P | P | I | N | N | I | A | I | A | P | I | P | A | P | P | I | P | I | N | A | A | A | I | A |
| Legha | 2020 | A | P | A | P | A | A | A | A | A | P | A | I | I | N | N | I | A | N | A | I | I | I | A | P | P | I | P | P | N | P | A | I | I | A |
| Liu | 2019 | A | P | A | P | A | A | P | A | I | P | P | I | I | N | N | P | A | N | I | I | I | I | A | A | A | I | P | I | N | P | A | I | I | P |
| Loades | 2020 | A | P | P | P | A | A | A | A | A | P | A | P | I | N | N | I | A | N | I | I | I | P | P | P | P | I | P | I | N | P | A | I | I | P |
| Lydsdottir | 2019 | A | P | A | A | A | A | I | A | I | P | A | A | P | N | N | P | A | N | I | I | I | I | A | A | A | A | P | P | N | A | A | I | I | A |
| Macêdo | 2018 | A | P | A | P | A | A | A | A | A | P | P | I | A | N | N | I | A | N | I | A | I | I | A | I | I | I | P | P | N | P | A | I | I | A |
| Marrie | 2018 | A | P | A | I | A | A | A | A | I | P | P | I | P | N | N | P | A | N | A | I | A | I | A | P | P | A | P | P | N | A | A | I | I | A |
| Martínez | 2020 | A | P | A | I | A | P | I | A | A | A | A | P | I | N | N | I | A | N | A | I | A | I | A | P | P | I | P | I | N | A | A | I | I | P |
| Massai | 2018 | A | P | A | P | A | A | A | P | A | P | P | I | I | N | N | I | A | N | I | I | I | I | A | P | P | I | P | I | N | P | A | I | I | I |
| Matthey | 2019 | A | P | A | P | A | P | P | P | A | P | P | A | I | N | N | I | P | N | I | I | I | I | A | P | P | A | P | I | N | P | A | I | I | A |
| McCartney | 2020 | A | P | A | P | A | I | I | A | I | P | P | I | P | N | N | P | A | N | I | I | I | I | A | A | A | I | P | P | N | P | A | I | I | A |
| Mohsin | 2021 | A | P | A | P | A | I | I | A | I | P | P | P | I | N | N | I | A | N | I | I | A | I | A | I | I | A | P | I | N | P | A | I | I | A |
| Molebatsi | 2020 | A | P | A | P | A | A | I | A | A | P | P | P | A | N | N | P | A | N | I | I | I | I | A | A | A | A | P | A | N | P | A | I | I | A |
| Muramatsu | 2018 | A | A | A | P | A | I | I | P | I | P | P | A | I | N | N | P | A | N | I | I | I | I | P | A | I | A | P | P | N | P | A | I | I | P |
| Nabbe | 2019 | A | P | A | P | A | A | I | A | I | A | P | I | I | N | N | I | A | N | I | I | A | P | P | I | I | A | P | I | N | A | A | I | I | P |
| Navarrete | 2019 | A | P | A | A | A | A | A | A | A | P | P | P | A | N | N | I | A | N | I | I | I | I | P | I | I | A | P | A | N | P | A | I | I | P |
| Park | 2020 | A | P | A | P | A | A | A | P | A | P | P | I | I | N | N | A | A | N | I | I | I | I | P | A | A | I | P | I | N | P | A | I | I | P |
| Peng | 2020 | A | P | A | P | A | A | I | A | I | P | P | P | I | N | N | I | A | N | I | I | I | I | A | P | P | A | P | I | N | I | A | I | I | P |
| Rancans | 2018 | A | P | A | P | A | A | A | P | A | A | A | I | I | N | N | P | A | N | P | I | I | I | A | I | I | A | P | I | N | P | A | I | I | P |
| Rashid | 2019 | A | A | A | P | A | A | I | P | I | P | P | I | I | N | N | I | A | N | I | I | I | I | A | I | I | I | P | P | N | P | A | I | I | I |
| Rashid | 2021 | A | P | P | I | A | A | I | P | I | P | P | I | A | N | N | P | A | N | I | I | I | I | A | I | I | I | P | P | N | P | A | I | I | P |
| Recklitis | 2020 | A | P | A | P | A | A | A | P | I | A | P | P | I | A | N | P | A | A | I | I | I | P | A | A | A | A | P | I | N | A | A | I | I | P |
| Risal | 2019 | A | P | P | P | A | A | I | A | A | P | P | I | A | N | I | P | A | N | I | I | I | I | P | P | P | I | A | P | N | P | A | I | I | P |
| Rodríguez-Mayoral | 2018 | A | P | A | P | A | A | A | A | A | P | P | I | I | N | N | P | A | N | I | I | A | I | A | P | P | A | P | I | N | I | A | I | I | I |
| Saal | 2018 | A | P | A | P | A | A | P | P | A | P | P | P | I | N | N | I | A | N | I | I | I | I | P | P | P | I | P | P | N | P | A | I | I | P |
| Saldivia | 2019 | A | P | A | P | A | A | I | P | A | P | P | P | I | N | N | I | A | N | I | I | I | I | P | P | P | I | P | I | N | P | A | I | I | A |
| Sampasa-Kanyinga | 2018 | A | P | A | A | A | I | I | A | I | A | P | I | I | N | N | I | A | N | A | I | P | I | A | A | P | I | P | P | N | P | A | I | I | A |
| Sasaki | 2019 | A | P | A | P | A | A | I | A | I | P | P | I | I | N | N | I | A | N | I | I | A | P | A | A | A | A | P | I | N | A | I | I | I | P |
| Scoppetta | 2021 | A | P | A | P | A | A | A | P | I | P | A | P | I | N | N | P | A | N | I | I | A | I | P | P | P | A | P | P | N | P | A | I | I | P |
| Searle | 2019 | A | P | A | P | A | A | A | A | A | A | A | P | A | N | N | A | A | N | P | I | I | I | A | P | P | A | P | A | N | A | A | I | I | P |
| Shaheen | 2019 | A | P | P | P | A | A | I | A | I | A | P | I | I | N | N | P | A | N | P | P | I | I | A | I | I | A | P | I | N | A | A | I | I | A |
| Shih | 2020 | A | P | A | P | A | A | I | A | I | P | P | P | I | N | N | I | A | N | I | I | I | I | A | P | P | I | P | I | N | P | A | I | I | P |
| Silagadze | 2019 | A | P | A | P | A | A | I | P | A | P | P | I | I | N | N | I | A | N | I | I | I | I | A | P | P | I | P | I | N | P | A | I | I | P |
| Smith Fawzi | 2019 | A | P | A | P | A | I | I | A | A | P | P | P | I | N | N | P | A | N | I | I | I | I | P | P | P | A | P | P | N | P | A | I | I | P |
| Smith-Nielsen | 2018 | A | P | A | A | A | A | A | A | I | P | A | I | I | N | N | P | A | N | I | I | I | I | A | A | I | A | P | P | N | P | A | I | I | A |
| Tan | 2018 | A | P | A | A | A | A | A | A | I | A | A | I | A | N | N | P | A | N | I | I | A | I | A | P | P | A | P | I | N | P | A | I | I | A |
| Tran | 2019 | A | P | A | P | A | I | I | A | A | A | A | P | A | N | N | I | P | N | P | I | I | I | A | P | P | I | I | I | N | P | A | I | I | A |
| Tschorn | 2019 | A | P | A | P | A | A | I | A | I | P | A | P | A | N | N | I | A | N | A | I | I | I | A | I | I | A | P | I | N | P | A | I | I | I |
| Udedi | 2019 | A | A | A | P | A | A | A | A | A | A | P | I | I | N | P | P | A | N | I | I | A | I | A | I | I | I | P | P | N | P | A | A | I | P |
| Urtasun | 2019 | A | P | A | P | A | A | A | A | A | P | P | A | I | N | N | P | A | N | I | I | A | I | P | P | P | I | P | I | N | P | A | I | I | A |
| van Heyningen | 2018 | A | P | P | I | A | A | A | P | A | A | P | P | I | N | I | I | A | N | I | I | I | I | A | A | A | I | P | I | N | P | A | I | I | A |
| van Heyningen | 2019 | A | A | A | I | A | A | A | A | A | P | P | P | I | N | N | I | A | N | I | I | I | I | A | I | I | A | P | I | N | P | A | I | I | P |
| Vázquez | 2019 | A | P | A | P | A | A | A | A | I | A | P | P | I | N | N | P | A | N | I | I | I | I | I | P | P | A | P | I | N | P | A | I | I | A |
| Vogeli | 2018 | A | P | A | P | A | A | A | P | I | P | P | I | I | N | N | I | A | N | I | P | I | I | A | I | I | A | P | I | N | I | A | I | I | P |
| Vrublevska | 2018 | A | P | A | A | A | A | A | P | A | A | P | A | P | N | N | I | A | N | I | P | I | I | A | A | A | A | P | P | N | A | A | I | I | P |
| Wang | 2018 | A | P | P | P | A | A | A | A | I | P | P | P | A | N | N | P | A | N | A | P | I | I | A | I | I | A | P | A | N | A | A | I | I | P |
| Wang | 2019 | A | P | A | I | A | A | I | A | I | P | P | I | I | N | N | P | A | N | P | I | I | I | A | A | A | A | P | I | N | A | A | I | I | P |
| Weihs | 2018 | A | P | A | P | A | A | I | A | A | P | P | I | I | N | I | I | A | N | A | I | I | I | A | A | A | I | P | P | N | A | A | I | I | A |
| Williams | 2020 | A | P | A | A | A | A | A | A | A | P | A | P | I | N | N | P | A | N | I | P | I | A | A | I | I | A | P | I | N | P | A | I | I | P |
| Williams | 2021 | A | P | I | P | A | A | P | P | I | A | P | I | P | N | N | I | A | N | I | I | I | I | A | I | I | I | P | P | N | A | A | I | I | A |
| Woldetensay | 2018 | A | P | A | P | A | A | I | P | A | P | P | I | I | N | N | A | A | N | I | I | I | I | A | A | A | I | P | I | N | P | A | I | I | A |
| Xia | 2019 | A | A | A | P | A | A | P | A | I | P | P | I | I | N | I | I | A | N | I | I | I | I | A | I | I | A | P | I | N | P | A | I | I | P |
| Xia | 2020 | A | P | A | P | A | A | A | A | I | P | P | P | I | N | I | I | A | N | I | I | I | I | A | I | I | I | P | I | N | P | A | I | I | P |
| Yang | 2018 | A | P | A | A | A | I | I | A | I | P | P | I | I | N | N | A | A | N | P | I | I | A | A | I | I | I | P | P | N | P | A | I | I | P |
| Yardeni | 2020 | A | P | A | P | A | A | A | A | I | P | P | I | I | N | N | P | A | N | I | I | I | P | A | I | I | I | P | I | N | A | A | I | I | P |
| Ye | 2020 | A | A | P | P | A | I | I | A | I | P | P | I | I | N | N | I | A | N | I | I | I | I | A | I | I | I | P | I | N | P | A | I | I | P |
| Yuan | 2019 | A | A | A | P | A | A | I | A | A | P | P | A | I | N | N | P | A | N | I | I | I | I | A | P | P | I | P | I | N | P | A | I | I | P |
| Zachar-Tirado | 2021 | A | P | A | A | A | A | A | A | A | P | P | I | A | N | N | I | A | I | I | I | I | I | A | P | P | I | P | P | N | A | A | I | I | P |
| Zinchuk | 2020 | A | P | A | P | A | A | A | P | I | P | P | I | I | N | N | I | A | N | I | I | I | I | A | A | A | I | P | P | N | P | A | I | I | P |

Abbreviations: A = Adequately reported; P = Partially reported; I = Inadequately or not reported; N = Not applicable.
